# The Use of Smartphone Keystroke Dynamics to Passively Monitor Upper Limb and Cognitive Function in Multiple Sclerosis: Longitudinal Analysis

**DOI:** 10.2196/37614

**Published:** 2022-11-07

**Authors:** Ka-Hoo Lam, James Twose, Birgit Lissenberg-Witte, Giovanni Licitra, Kim Meijer, Bernard Uitdehaag, Vincent De Groot, Joep Killestein

**Affiliations:** 1 Department of Neurology Amsterdam University Medical Centers (VU University Medical Center location) Amsterdam Netherlands; 2 Neurocast BV Amsterdam Netherlands; 3 Department of Epidemiology and Data Science Amsterdam University Medical Centers (VU University Medical Center location) Amsterdam Netherlands; 4 Department of Rehabilitation Medicine Amsterdam University Medical Centers (VU University Medical Center location) Amsterdam Netherlands

**Keywords:** multiple sclerosis, smartphone, mobile app, digital technology, keystroke dynamics, typing, upper extremity, cognition, outpatient monitoring

## Abstract

**Background:**

Typing on smartphones, which has become a near daily activity, requires both upper limb and cognitive function. Analysis of keyboard interactions during regular typing, that is, keystroke dynamics, could therefore potentially be utilized for passive and continuous monitoring of function in patients with multiple sclerosis.

**Objective:**

To determine whether passively acquired smartphone keystroke dynamics correspond to multiple sclerosis outcomes, we investigated the association between keystroke dynamics and clinical outcomes (upper limb and cognitive function). This association was investigated longitudinally in order to study within-patient changes independently of between-patient differences.

**Methods:**

During a 1-year follow-up, arm function and information processing speed were assessed every 3 months in 102 patients with multiple sclerosis with the Nine-Hole Peg Test and Symbol Digit Modalities Test, respectively. Keystroke-dynamics data were continuously obtained from regular typing on the participants’ own smartphones. Press-and-release latency of the alphanumeric keys constituted the fine motor score cluster, while latency of the punctuation and backspace keys constituted the cognition score cluster. The association over time between keystroke clusters and the corresponding clinical outcomes was assessed with linear mixed models with subjects as random intercepts. By centering around the mean and calculating deviation scores within subjects, between-subject and within-subject effects were distinguished.

**Results:**

Mean (SD) scores for the fine motor score cluster and cognition score cluster were 0.43 (0.16) and 0.94 (0.41) seconds, respectively. The fine motor score cluster was significantly associated with the Nine-Hole Peg Test: between-subject β was 15.9 (95% CI 12.2-19.6) and within-subject β was 6.9 (95% CI 2.0-11.9). The cognition score cluster was significantly associated with the Symbol Digit Modalities Test between subjects (between-subject β –11.2, 95% CI –17.3 to –5.2) but not within subjects (within-subject β –0.4, 95% CI –5.6 to 4.9).

**Conclusions:**

Smartphone keystroke dynamics were longitudinally associated with multiple sclerosis outcomes. Worse arm function corresponded with longer latency in typing both across and within patients. Worse processing speed corresponded with higher latency in using punctuation and backspace keys across subjects. Hence, keystroke dynamics are a potential digital biomarker for remote monitoring and predicting clinical outcomes in patients with multiple sclerosis.

**Trial Registration:**

Netherlands Trial Register NTR7268; https://trialsearch.who.int/Trial2.aspx?TrialID=NTR7268

## Introduction

In multiple sclerosis (MS), a vast number of disease-modifying therapies targeting disease activity are available, and therapies preventing (and potentially counteracting) disease progression are emerging [1-3]. Additional treatment modalities include nonpharmacological therapies, such as rehabilitative and cognitive therapies [4,5]. This wide array of expanding treatment options will increasingly lead to patient-centered disease management. The personalized treatment of MS would strongly benefit from early and improved recognition of disability progression or symptom onset. However, disease progression (ie, deterioration of neurological function independent of relapses) and newly occurring symptoms are often subtle in MS [6]. Additionally, the currently most widely used clinical measure in MS, the Expanded Disability Status Scale (EDSS) [7], assesses neurological function over a period spanning a year or almost a year and may need reassessment over time to confirm deterioration [6]. The Multiple Sclerosis Functional Composite (MSFC) consists of brief objective measurements in 3 important domains in MS: ambulatory, upper limb, and cognitive function. It was designed to complement the EDSS and improve sensitivity in capturing disease status [8]. Compared to the extensive implementation of the MSFC in clinical trials, it has been poorly incorporated into clinical practice, as clinical evaluations are too sporadic for the measure to be sensitive or provide meaningful temporal information for monitoring patients on the individual level [9].

The advent of digital devices allows for more continuous and more fine-grained measurements of biometrics that could be related to functioning in patients with MS. With the digitalization of society, smartphones have become widespread and part of everyday living. Consequently, keystroke dynamics (KD) from typing on smartphones has been investigated for quantifying disability in MS. KD encompasses quantitative metrics of keyboard interactions during regular typing. In our previous work, KD was found to be correlated with upper limb and cognitive function, and, to a lesser extent, overall disability, as measured with the EDSS [10]. Across a wide range of KD features and aggregation methods, KD was also found to reach adequate responsiveness to meaningful change in radiological disease activity, ambulatory function, and upper limb function over a period of 3 months [11]. Additionally, analysis of KD data using a nonlinear time-series approach identified potential indicators of clinical change [12]. Based on these previous findings, 2 keystroke clusters were derived, one specific to upper limb function and the other to cognition, since these 2 domains are most directly related to typing. In order to translate this new biomarker into clinical practice for monitoring upper limb function and cognition in MS, the association with clinical measures over time and within individual patients needs investigation.

Our objective was to investigate the longitudinal associations between KD features, passively derived from regular typing on a smartphone, and upper limb function and cognition in patients with MS. Additionally, we sought to differentiate these longitudinal associations for both between-subject differences and within-subject changes in order to enable disease monitoring on the individual-patient level.

## Methods

### Study Design and Participants

This was a prospective cohort study at the MS Center of the Amsterdam University Medical Centers (VU University Medical Center location). The study design and interim analyses have been reported previously [10,11]. In brief, after a baseline assessment (M_0_), we followed the patients for 1 year, with clinical visits every 3 months (M_3_, M_6_, M_9_, and M_12_). During the study, participants used the Neurokeys keyboard app on their own smartphones [13]. Participants were patients with MS and were consecutively included in the study between August 2018 and December 2019 until a cohort size of 100 participants was reached. Patients were eligible if they were aged between 18 and 65 years, had a definite diagnosis of MS, had an EDSS score below 7.5, had access to a smartphone with the Android (5.0 or higher) or iOS (10 or higher) operating systems, had no visual or upper extremity deficits affecting regular smartphone use, and had no mood or sleep disorders impacting daily living (based on medical history-taking by a screening physician).

### Ethical Considerations

The study received ethical approval from the Medisch Ethische Toetsingscommissie Vrije Universiteit medisch centrum (reference 2017.576) and conformed to legislation regarding data privacy and medical devices (Dutch Health and Youth Care Inspectorate; reference VGR2006948). All patients gave written informed consent. The study was registered as trial number NTR7268 at the Netherlands Trial Register.

### Clinical Outcomes

Clinical outcomes for important aspects of MS were assessed, including clinically reported relapses, conventional magnetic resonance imaging (MRI) for disease activity, the EDSS, the MSFC, patient-reported outcomes, quantitative MRI, and optical coherence tomography for evaluation of domain-specific and overall disease severity and disease progression over time. As KD is most directly related to upper limb function and cognition, the current analysis focuses on the clinical assessments made every 3 months with the Nine-Hole Peg Test (NHPT) and Symbol Digit Modalities Test (SDMT). The NHPT is a measure of upper limb function that records the time needed to place, with a single hand, 9 pegs into 9 holes and then remove them [14]. The task is performed twice for each hand, and the 4 trials are averaged into a single score, with a higher score reflecting worse performance. The SDMT is a measure of information processing speed, the cognitive domain that is most commonly affected in MS and indicates overall cognitive functioning [15]. Using a key with 9 symbol-digit pairings, the number of correct digits corresponding to symbols during a 90-second trial is recorded as the total score [16]. A higher score reflects better performance.

### KD and Keystroke Features

**Figure 1:**
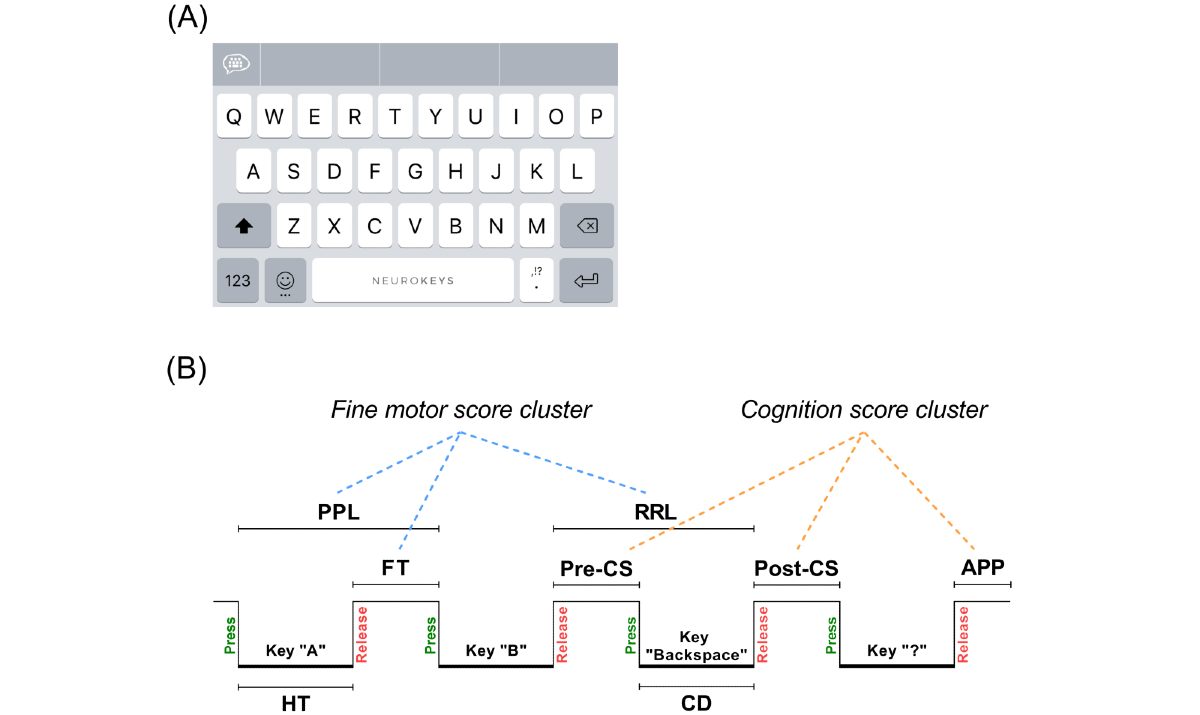
Overview of the Neurokeys keyboard (A) and a schematic representation of the keystroke dynamics features and clusters (B). APP: after-punctuation pause; CD: correction duration; FT: flight time; HT: hold time; post-CS: postcorrection slowing; PPL: press-press latency; pre-CS: precorrection slowing; RRL: release-release latency.

During the 1-year follow-up period, patients used the Neurokeys app (Neurocast BV) on their own smartphones [13]. The Neurokeys app replaces the native keyboard with a similar-looking keyboard (Figure 1A) that passively and continuously collects data on press-and-release typing events during everyday typing. From these keyboard interactions, keystroke features are derived based on key type (Figure 1B). For alphanumeric keys, the features include the latency between presses (press-press latency) and releases (release-release latency), the keypress time (hold time), and the time between keys (flight time). For the backspace key, derived features include latency prior to the use of the key (precorrection slowing), during use (correction duration) and after use (postcorrection slowing). Lastly, the time after a punctuation key was used was also derived (after-punctuation pause). A keystroke event count threshold of 50 events was used to remove days with insufficient data.

### Construction of Keystroke Clusters

To compare the continuously collected keystroke data with clinical outcomes, the keystroke features were aggregated and clustered. First, the keystroke features were aggregated per day by the mean and median values, as both statistical measures summarize the data well and remain on the same unit scale (ie, seconds) to retain interpretability. Since mean and median values of the keystroke features were highly correlated, rather than discarding one, both summary values were averaged to reduce potential multicollinearity. Second, the fine motor score cluster (FMSC) and cognition score cluster (CSC) were derived based on the hypothesis that timing-related features (press-press latency, release-release latency, hold time, and flight time) are more related to fine motor skills, while error-related (precorrection slowing, correction duration, and postcorrection slowing) and paralinguistic (after-punctuation pause) features are more specific to events reflecting cognitive function. This concept-based clustering was then analyzed with principal component analysis and correlation analysis (Multimedia Appendices 1 - 3). Only features that contributed equally in the component analysis and were highly correlated (*r*>0.50) were included in the final cluster [17]. Finally, near the time of each clinical visit, 28-day (the 14 days before and after the clinical visit) and 14-day (the 7 days before and after the clinical visit) aggregation periods for the keystroke clusters was chosen for FMSC and CSC, respectively, since fine motor function can be considered more stable over time than cognitive function. The 28-day and 14-day periods for the keystroke clusters were aggregated by the mean value. Using these criteria, FMSC included press-press latency, release-release latency, and flight time, whereas CSC included precorrection slowing, postcorrection slowing, and after-punctuation pause.

### Statistical Analysis

Analysis was performed with SPSS (version 26; IBM Corp) and R (version 4.0.3; R Foundation for Statistical Computing). Categorical data were summarized as the frequency and percentage. Numerical data were summarized as the mean and SD (or median and IQR or range if normally distributed). A linear mixed model analysis was used to determine the longitudinal association between KD clusters and clinical outcomes, so as to take into account clustering of repeated measurements within subjects [18]. Separate intercepts were estimated for each subject, over which a normal distribution was drawn. Then, the variance was estimated from that normal distribution and added to the model as a random intercept (ε), to adjust for repeated measurements within subjects, as follows: Y = β_0_ + β_1_X + ε. For upper limb function, the dependent variable was the NHPT score, the independent variable was FMSC, and the covariates were age and sex. For information processing speed, the dependent variable was the SDMT score, the independent variable was CSC, and the covariates were age, sex, and level of education. Since there was a significant relationship between time and SDMT performance, most likely due to practice effects, an additional random intercept for time (in days) was added to the cognition model. This allowed varying intercepts based on time in order to account for practice effects and imbalances in time intervals between clinical visits across subjects [19].

Importantly, given that the effect estimates of a linear mixed model analysis in a cohort with repeated measures are overall effects (ie, effect estimates entangle both differences across subjects and changes within subjects over time), a “hybrid” linear mixed model analysis was performed to disentangle the between-subject and within-subject effects of the longitudinal association [20]. This was done by centering around the mean and calculating deviation scores at each clinical visit for each subject. The mixed model analysis was then performed with both the centered values and the deviation score of this centered value for each individual, as follows: 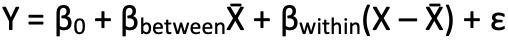, where β_between_ is the between-subject effect and β_within_ is the within-subject effect [18].

The output of all linear mixed models included effect estimates, 95% CIs, *P* values, and percentage explained variance. Covariates were considered relevant if the effect estimate between the dependent and independent variables changed by 10% or more after including the covariates into the model [21].

## Results

**Figure 2:**
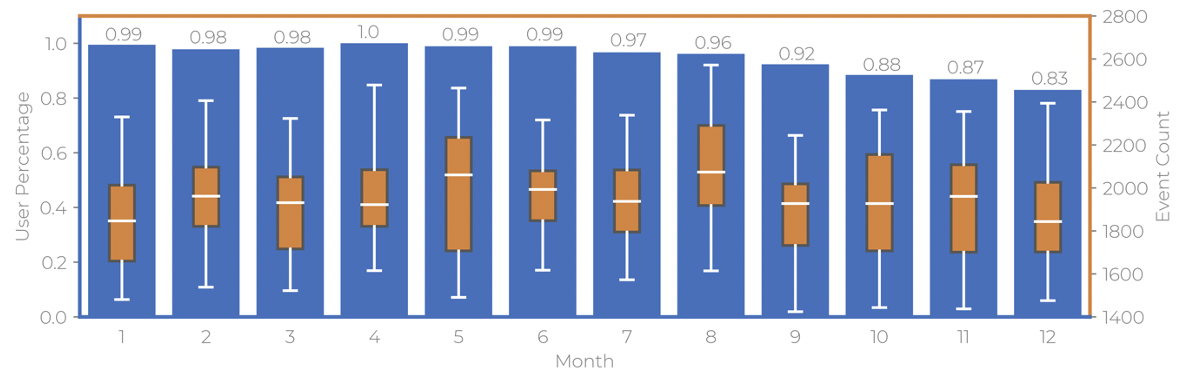
Bar graph depicting the retention rate (left y-axis, “user percentage”) of patients per month with superimposed box plots of the number of daily keystroke events (right y-axis, “event count”). The values above the bars show the retention rates as percentages.

A total of 102 patients with MS were included, of whom 91 completed the follow-up at M_12_; 6 patients dropped out at M_3_, 1 at M_6_, 1 at M_9_, and 3 at M_12_. The demographic and clinical characteristics at baseline are summarized in Table 1. The patients had a mean age of 46.4 years, most were female (75/102, 73.5%), and most had the relapsing-remitting MS subtype (61/102, 59.8%). The median disease duration since diagnosis was 5.7 years and the median EDSS score was 3.5. The mean follow-up duration was 376.9 (SD 109.4) days. At M_12_, the retention rate of patients with active keyboard use was 83.3% (85/102). Figure 2 shows the monthly retention rate and the average number of keystroke events per day. The clinical outcomes per visit and keystroke cluster data corresponding with each clinical visit are summarized in Table 2 and Multimedia Appendices 4 and 5. Part of the study follow-up coincided with the COVID-19 pandemic, which resulted in missing clinical visits, most prominently at M_6_ and M_9_.

**Table 1:** Baseline patient demographic and clinical characteristics.

Characteristics			Patients with multiple sclerosis (N=102)
Age (years), mean (SD)			46.4 (10.4)
**Sex, n (%)**			
	Female	75 (73.5)	
	Male	27 (26.5)	
**Education level^a^, n (%)**			
	Low	3 (2.9)	
	Middle	34 (33.3)	
	High	65 (63.7)	
**Multiple sclerosis type, n (%)**			
	Primary progressive	11 (10.8)	
	Secondary progressive	30 (29.4)	
	Relapsing remitting	61 (59.8)	
Disease duration since diagnosis (years), median (IQR)			5.7 (3.0-13.1)
Expanded Disability Status Scale score, median (range)			3.5 (1.5-7.0)

^a^Education levels were defined according to Rijnen et al [22].

**Table 2:** Clinical outcomes and keystroke dynamics clusters for each clinical visit.

Outcomes		M_0_	M_3_	M_6_	M_9_	M_12_
**Nine-Hole Peg Test^a^**						
	Subjects, n	102	93	76	58	89
	Time (seconds), median (IQR)	21.2 (19.4-25.0)	21.0 (18.7-24.0)	20.5 (18.8-22.5)	20.2 (18.5-22.0)	20.3 (18.7-23.0)
**Symbol Digit Modalities Test**						
	Subjects, n	102	93	76	58	90
	Mean score (SD)	54.4 (10.3)	56.8 (10.4)	57.9 (12.0)	61.3 (12.8)	60.3 (12.9)
**Fine motor score cluster**						
	Subjects, n	96	88	72	55	71
	Time (seconds), mean (SD)	0.45 (0.16)	0.44 (0.16)	0.44 (0.17)	0.39 (0.15)	0.42 (0.17)
	Days^b^ (n), mean (SD)	14.3 (2.4)	26.4 (4.8)	26.2 (4.8)	25.5 (5.4)	15.5 (5.5)
**Cognition score cluster**						
	Subjects, n	101	89	70	55	72
	Time (seconds), mean (SD)	1.01 (0.42)	0.95 (0.40)	0.92 (0.40)	0.86 (0.38)	0.90 (0.44)
	Days^b^ (n), mean (SD)	7.8 (1.0)	13.7 (1.0)	13.3 (2.2)	13.1 (2.1)	8.4 (2.7)

^a^For the Nine-Hole Peg Test, an average outlier threshold of 40 seconds was implemented, excluding 14 of 387 samples (3.6%).

^b^Only days with ≥50 keystroke events.

### Upper Limb Function

**Figure 3:**
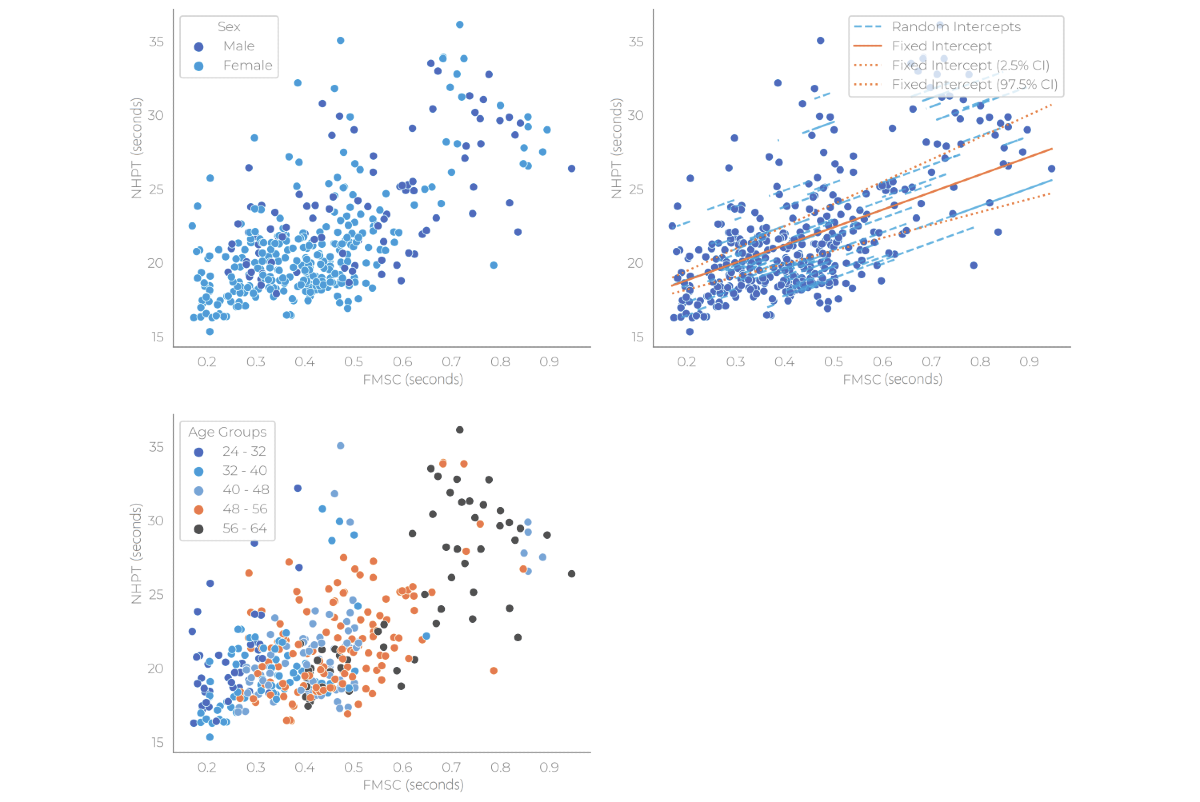
Scatter plots and linear mixed model fit for the Nine-Hole Peg Test and fine motor score cluster by covariates (sex and age), with random intercepts on subject level, and the number of days that constituted the keystroke cluster data points. FMSC: fine motor score cluster; NHPT: Nine-Hole Peg Test.

For the association between the NHPT and FMSC, 98 patients with MS were included in the mixed model analysis with an average of 3.9 observations per patient. Overall, the mean (SD) for FMSC was 0.43 (0.16) seconds and the median (IQR) for the NHPT was 20.6 (18.8-23.3) seconds. The results of the mixed model analysis are shown in Table 3 and depicted visually in Figure 3. In the overall model, FMSC was significantly associated with the NHPT and explained 42% of the variance in the NHPT results. Age and sex were not found to be relevant confounders in this association. In the hybrid model, a one-SD (0.16-second) increase in FMSC was significantly associated with an increase in NHPT of 2.5 seconds between patients and 1.1 seconds within patients.

**Table 3:** Results of linear mixed model analyses of Nine-Hole Peg Test results over time with a random intercept on subject level.

		*β* (95% CI)	*P* value	Random effect variance, %		Explained variance, %	
Intercept only					13.7		N/A^a^
Fine motor score cluster		12.62 (9.61-15.63)	<.001	8		42	
Fine motor score cluster and covariates^b^		12.56 (8.96-16.16)	<.001	7.7		43.9	
**Hybrid model**					7.7		43.7
	Between subjects	15.91 (12.18-19.63)	<.001	N/A		N/A	
	Within subjects	6.94 (2.00-11.87)	<.001	N/A		N/A	

^a^N/A: not applicable.

^b^Covariates included age and sex.

### Information Processing Speed

**Figure 4:**
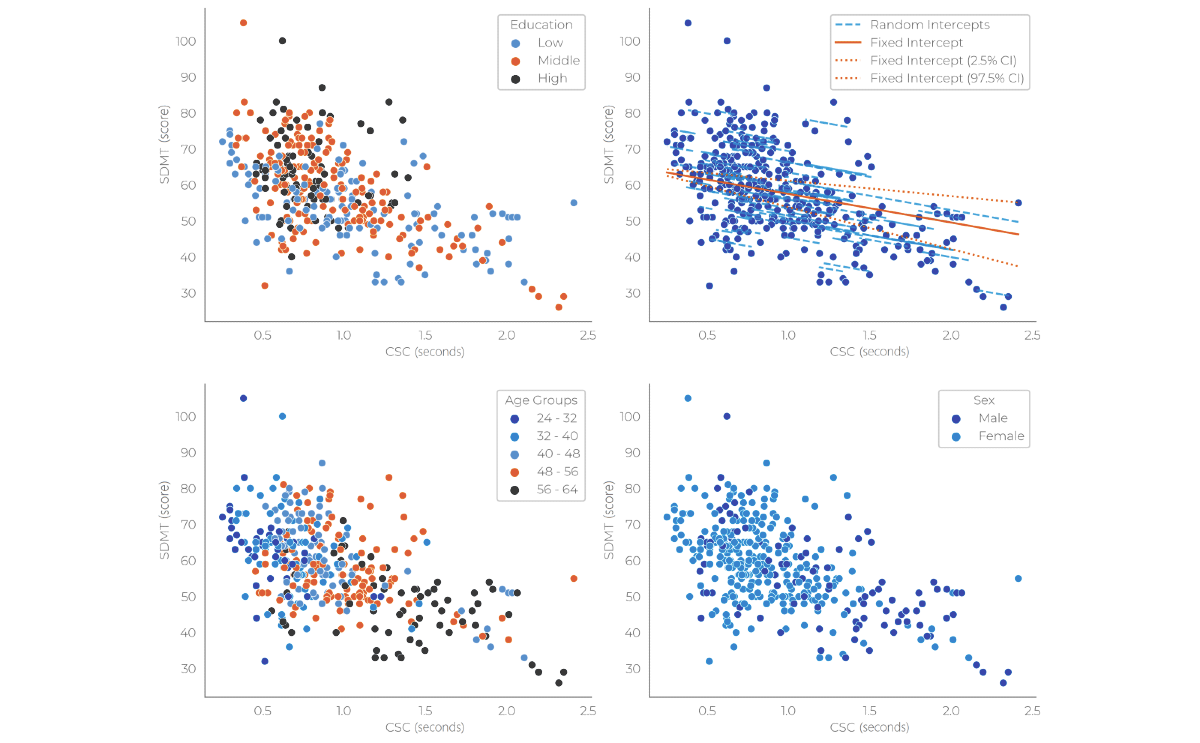
Scatter plots and linear mixed model fit for Symbol Digit Modalities Test and cognition score cluster by covariates (level of education, age, and sex) and random intercepts on subject level. SDMT: symbol digit modalities test; CSC: cognition score cluster.

All 102 patients with MS were included in the analysis of the association between information processing speed and the cognition keystroke cluster. The patients had an average of 3.8 repeated observations. The overall mean (SD) was 0.94 (0.41) seconds for CSC and 58.9 (12.1) points for SDMT. The output of the mixed model analyses is summarized in Table 4 and shown visually in Figure 4. In the overall model, CSC was significantly associated with SDMT and, together with age, sex, and level of education, explained 30.4% of the variance in SDMT. In the hybrid model, an increase of 1 SD (0.41 seconds) in CSC was significantly associated with a decrease of –4.6 in SDMT between patients. The within-subject association between CSC and SDMT, however, was not statistically significant.

**Table 4:** Results of linear mixed model analyses of the Symbol Digit Modalities Test results over time with random intercepts on subject level and time (in days).

			*β* (95% CI)		*P* value		Random effect variance, %			Explained variance, %	
Intercept only								110.9			N/A^a^
Cognition score cluster		–8.57 (–12.02 to –5.12)		<.001		82.7			25.4		
Cognition score cluster and covariates^b^		–5.02 (–9.02 to –1.02)		.02		77.1			30.4		
**Hybrid model (including covariates^a^)**								74.4			32.9
	Between subjects	–11.25 (–17.28 to –5.21)		<.001		N/A			N/A		
	Within subjects	–0.35 (–5.60 to 4.89)		.9		N/A			N/A		

^a^N/A: not applicable.

^b^Covariates included age, sex, and level of education.

## Discussion

### Principal Findings

This study investigated the longitudinal association between smartphone KD and commonly used clinical measures for upper limb function and information processing speed in patients with MS. In the overall model, the fine motor keystroke cluster was significantly associated with the NHPT (β=12.6, 95% CI 9.6-15.6); higher latency for presses and releases of alphanumeric keys during typing was related to a worse performance on the NHPT. When splitting the model for between-subject and within-subject effects, the association remained significant for both (β=15.9, 95% CI 12.2-19.6, and β=6.9, 95% CI 2.0-11.9, respectively). For the association between the cognitive keystroke cluster and SDMT, the time in days was included to account for practice effects on the SDMT and the imbalance in intervals between visits across subjects. CSC was found to be significantly negatively associated with SDMT; higher latency for backspace and punctuation mark keypresses was related to a worse SDMT score. This association had a β of –5.0 (95% CI –9.0 to –1.0) after adjusting for age, sex, and level of education. In the hybrid model for the cognitive keystroke cluster, the between-subject effect increased to β=–11.2 (95% CI –17.3 to –5.2), whereas the within-subject effect decreased to β=–0.4 (95% CI –5.6 to 4.9). To improve the interpretability of these associations, rather than considering 1-unit changes in keystroke clusters, the effect sizes can be recalculated to represent a change of 1 SD in keystroke clusters. In this distribution-based approach, a 1-SD change in FMSC corresponded with a change in NHPT of 1.1 seconds within patients or a 2.5-second difference between patients. Likewise, a 1-SD change in CSC corresponded with a change in SDMT of –0.14 points (although this was not significant) within patients or a –4.6-point difference between patients. Therefore, in our current cohort, a 2-SD change in FMSC and a 1-SD change in CSC would correspond to clinically relevant changes, as a 20% change in NHPT and a 4-point change in SDMT are considered clinically relevant based on group studies [14,16].

### Comparison With Prior Work

Measurements of task or activity performance are an integral part of assessing and monitoring chronic neurological disorders such as MS. Typing on a smartphone is a near-daily activity from which biometric information pertaining to physical or mental functions can be derived. Despite this, the use of KD in the assessment of diseases is relatively underutilized, especially considering that touchscreen typing has existed for over a decade. Hence, our objective was to validate the use of passive KD, measured with the Neurokeys app, to improve disease management in MS. To this end, earlier investigations by our research group reported on the clinimetric properties of reliability, validity, and 3-month responsiveness of KD in MS [10,11]. To the best of our knowledge, other applications of KD analysis in diseases are limited to the detection of early-stage Parkinson disease [23-28], upper limb dysfunction in amyotrophic lateral sclerosis [29], and severity in mood disorders [30,31]. The objective of the studies of Parkinson disease was to differentiate subjects with disease or early disease from those without, making the study endpoints not directly comparable to ours. Nevertheless, the study on amyotrophic lateral sclerosis found worse typing to be associated with progression of the disease, which is similar to our current findings. Last, the 2 studies on mood disorders found significant regression effects between severity of depressive symptoms and smartphone keyboard activity. This is in line with our findings, in which worse typing parameters corresponded to worse performance on the clinical tests. In addition, concurrent to our findings, these studies showed that KD can be utilized and can even outperform clinical standards in the detection and assessment of disease status through capitalizing on motor anomalies and, to some extent, cognitive dynamics that affect typing behavior.

Of note is that, besides our 3-month responsiveness interim analysis, there are currently no studies investigating KD in a longitudinal setting in patients with MS. While research investigating differences between subjects is of great importance, especially in early validation research, differences across subjects cannot directly be extrapolated to changes that occur within individuals over time. Therefore, analyzing change over time within patients is essential for monitoring or predictive modeling in MS. Splitting our model to separately determine between-subject and within-subject effects showed that the latter were stronger than the former. This suggests that in our sample, differences in upper limb function and information processing speed tended to be greater across patients than within patients, as shown by the SD of the outcomes across patients being much larger than the average change over time. This is not surprising, given that research in outcome measures in MS often struggles to achieve adequate sensitivity to change over time compared to correlations across patients [32]. For upper limb function, our model that separated the between-subject and within-subject effects still showed a strong, significant within-subject effect estimate, indicating that the fine motor keystroke cluster is sensitive to change within individuals.

For information processing speed, prior to adjustment for time, we also found a significant within-subject effect estimate for the cognitive keystroke cluster (data not shown). However, accounting for practice effects by adding the time point as a random effect to the model resulted in a lower, statistically nonsignificant within-subject effect. This suggests that the association between SDMT and the cognitive keystroke cluster in our current cohort was affected more strongly by the effect of learning than by changes in the keystroke cluster within patients. This explanation is supported by the results of modeling the effect of time as a fixed term instead of a random effect. In this model, the effect of time was stronger than the within-subject effect of the cognition keystroke cluster. In addition, when time as a fixed term was modeled categorically (such as M_0_, M_3_, or M_6_), instead of being linear, the effect of time on the association between SDMT and cognition keystroke cluster was larger at later time points than earlier time points. As the amount of learning differs between patients, patients who are less severely affected by MS tend to have stronger practice effects than patients with more severe disability [33], and the larger positive slopes at later time points can be explained by practice effects causing a larger spread in SDMT data over time while the cognition keystroke cluster stays more or less stable.

Despite practice effects most likely diluting our findings on the within-subject association, the strong between-subject effect demonstrates the promise of the use of KD as a biomarker of information processing speed. Therefore, monitoring of cognition using KD needs further investigation with clinical cognitive outcomes that are more sensitive or less affected by practice effects and with a study population that allows a closer focus on cognitive function (ie, by including the presence of cognitive deficits as a selection criterion or using a longer follow-up duration) to demonstrate effects larger than measurement variability or learning effects. Similarly, a smartphone-based cognition test in the same cohort was found to be valid on the cross-sectional level, but lacked responsiveness when looking at change longitudinally, as changes within subjects are subtler than differences across subjects and measurements can be variable [34]. Additionally, more advanced analysis methods, such as nonlinear models, may increase sensitivity and allow higher frequency keystroke data and further investigation [12,35].

### Limitations

A few limitations should be considered. First, despite modeling time in the analyses to take into account score changes over time, practice effects were not adjusted for, such by having healthy controls throughout the study. In addition, practice effects may have been exacerbated in the current cohort by their weekly performance of a smartphone variant of the SDMT concurrently with the digital biomarkers. Second, we investigated 2 commonly affected domains in MS that are directly involved with typing on the smartphone: upper limb function and information processing speed. In reality, MS entails a much broader array of functional spheres and relevant treatment outcomes. We collected a broad scope of clinical outcomes in our current cohort, and these data should be examined in future work in order to incorporate KD as a complete tool for monitoring MS. Lastly, a significant number of patients had missing clinical data, most prominently at M_6_ and M_9_, due to the COVID-19 pandemic. This also created a bias, as patients with secondary or primary progressive MS missed their clinical visits more often than patients with relapsing-remitting MS.

### Conclusions

Keystroke clusters constructed from passively acquired smartphone KD data were shown to reflect function in patients with MS in a longitudinal setting, as measured with commonly used clinical outcome measures of upper limb function and cognitive functioning. In the longitudinal repeated measures analysis, the fine motor keystroke cluster was found to be associated with upper limb function across and within patients. This attests to the use of KD for monitoring and predictive purposes in MS. Monitoring cognitive function with KD needs further investigation, as a significant association was found in the overall model, but this relied mostly on differences between patients rather than changes within patients, likely exacerbated by practice effects related to the clinical measures. Altogether, KD during typing provided detailed data on the temporal and granular level on everyday upper limb and cognitive function. Our current findings are the first to demonstrate associations between clinical outcomes in MS and smartphone typing performance. With the ongoing expansion of therapeutic interventions, KD as a remote passive biomarker may improve clinical assessment and patient-centered disease management in MS. Important steps for future research are investigating other highly relevant MS outcomes, such as disease activity, and the external validity of the current results by monitoring function in clinical practice on the individual-patient level.

## Appendix

Multimedia Appendix 1 Supplementary table 1. Principal component analysis of the timing-related and error-related/paralinguistic keystroke features.

Multimedia Appendix 2 Supplementary table 2. Correlation matrix of the timing-related keystroke features.

Multimedia Appendix 3 Supplementary table 3. Correlation matrix of the error-related/paralinguistic keystroke features.

Multimedia Appendix 4 Supplementary figure 1. Line graph of individual patients’ NHPT scores during the study.

Multimedia Appendix 5 Supplementary figure 2. Line graph of individual patients’ SDMT scores during the study.

## Abbreviations

CSC: cognition score cluster
EDSS: Expanded Disability Status Scale
FMSC: fine motor score cluster
KD: keystroke dynamics
MRI: magnetic resonance imaging
MS: multiple sclerosis
MSFC: Multiple Sclerosis Functional Composite
NHPT: Nine-Hole Peg Test
SDMT: Symbol Digit Modalities Test

## Data Availability

Anonymized data not published within the article are available upon request from a qualified investigator. Such requests must be submitted in writing and will be reviewed for researcher qualifications and the legitimacy of the research purpose.
