# Supplementary material for: The Use of Smartphone Keystroke Dynamics to Passively Monitor Upper Limb and Cognitive Function in Multiple Sclerosis: Longitudinal Analysis
Source: J Med Internet Res. 2022 Nov 7;24(11):e37614. doi: 10.2196/37614 (PMC9679948; doi:10.2196/37614)
Supplement: Multimedia Appendix 1 [file jmir_v24i11e37614_app1.docx]

**Supplementary table 1.** Principal component analysis of the timing-related and error-related/paralinguistic keystroke features

| Timing-related keystroke features | Principal component 1 (explained variance=77.9%) | Principal component 1, without HT (explained variance=98.5%) |
| --- | --- | --- |
| FT_mean | 0.391 | 0.408 |
| PPL_mean | 0.396 | 0.408 |
| RRL_mean | 0.396 | 0.408 |
| FT_median | 0.388 | 0.407 |
| PPL_median | 0.395 | 0.409 |
| RRL_median | 0.395 | 0.408 |
| HT_mean | 0.194 |  |
| HT_median | 0.180 |  |
|  |  |  |
| Error-related/paralinguistic keystroke features | Principal component 1 (explained variance=57.7%) | Principal component 1, without CD (explained variance=73.4%) |
| Pre-CS_mean | 0.413 | 0.423 |
| Post-CS_mean | 0.419 | 0.428 |
| APP_mean | 0.354 | 0.368 |
| Pre-CS_median | 0.416 | 0.430 |
| Post-CS_mean | 0.404 | 0.418 |
| APP_median | 0.360 | 0.377 |
| CD_mean | 0.238 |  |
| CD_median | 0.073 |  |

Abbreviations: FT, flight time; PPL, press-press latency; RRL, release-release latency; HT, hold time; Pre-CS, pre-correction slowing; Post-CS, post-correction slowing; APP, after punctuation pause; CD, correction duration.
