# Supplementary material for: The Use of Smartphone Keystroke Dynamics to Passively Monitor Upper Limb and Cognitive Function in Multiple Sclerosis: Longitudinal Analysis
Source: J Med Internet Res. 2022 Nov 7;24(11):e37614. doi: 10.2196/37614 (PMC9679948; doi:10.2196/37614)
Supplement: Multimedia Appendix 2 [file jmir_v24i11e37614_app2.docx]

**Supplementary table 2.** Correlation matrix of the timing-related keystroke features

|  | PPL_mean | PPL_median | RRL_mean | RRL_median | FT_mean | FT_median | HT_mean | HT_median |
| --- | --- | --- | --- | --- | --- | --- | --- | --- |
| PPL_mean | 1.000 |  |  |  |  |  |  |  |
| PPL_median | 0.966 | 1.000 |  |  |  |  |  |  |
| RRL_mean | 1.000 | 0.966 | 1.000 |  |  |  |  |  |
| RRL_median | 0.965 | 0.999 | 0.965 | 1.000 |  |  |  |  |
| FT_mean | 0.995 | 0.961 | 0.995 | 0.960 | 1.000 |  |  |  |
| FT_median | 0.953 | 0.991 | 0.953 | 0.991 | 0.960 | 1.000 |  |  |
| HT_mean | 0.341 | 0.332 | 0.342 | 0.331 | 0.266 | 0.231 | 1.000 |  |
| HT_median | 0.278 | 0.264 | 0.279 | 0.261 | 0.202 | 0.162 | 0.986 | 1.000 |

Abbreviations: PPL, press-press latency; RRL, release-release latency; FT, flight time; HT, hold time.
