# Supplementary material for: The Use of Smartphone Keystroke Dynamics to Passively Monitor Upper Limb and Cognitive Function in Multiple Sclerosis: Longitudinal Analysis
Source: J Med Internet Res. 2022 Nov 7;24(11):e37614. doi: 10.2196/37614 (PMC9679948; doi:10.2196/37614)
Supplement: Multimedia Appendix 3 [file jmir_v24i11e37614_app3.docx]

**Supplementary table 3.** Correlation matrix of the error-related/paralinguistic keystroke features

|  | Pre-CS_mean | Pre-CS_median | Post-CS_mean | Post-CS_median | APP_mean | APP_median | CD_mean | CD_median |
| --- | --- | --- | --- | --- | --- | --- | --- | --- |
| Pre-CS_mean | 1.000 |  |  |  |  |  |  |  |
| Pre-CS_median | 0.972 | 1.000 |  |  |  |  |  |  |
| Post-CS_mean | 0.847 | 0.841 | 1.000 |  |  |  |  |  |
| Post-CS_median | 0.867 | 0.887 | 0.927 | 1.000 |  |  |  |  |
| APP_mean | 0.545 | 0.535 | 0.564 | 0.555 | 1.000 |  |  |  |
| APP_median | 0.592 | 0.599 | 0.603 | 0.620 | 0.811 | 1.000 |  |  |
| CD_mean | 0.330 | 0.272 | 0.452 | 0.364 | 0.284 | 0.272 | 1.000 |  |
| CD_median | -0.130 | -0.139 | 0.012 | -0.023 | -0.003 | -0.052 | 0.645 | 1.000 |

Abbreviations: Pre-CS, pre-correction slowing; Post-CS, post-correction slowing; APP, after punctuation pause; CD, correction duration.
