# Supplementary figures and images for: The Use of Smartphone Keystroke Dynamics to Passively Monitor Upper Limb and Cognitive Function in Multiple Sclerosis: Longitudinal Analysis
Source: J Med Internet Res. 2022 Nov 7;24(11):e37614. doi: 10.2196/37614 (PMC9679948; doi:10.2196/37614)

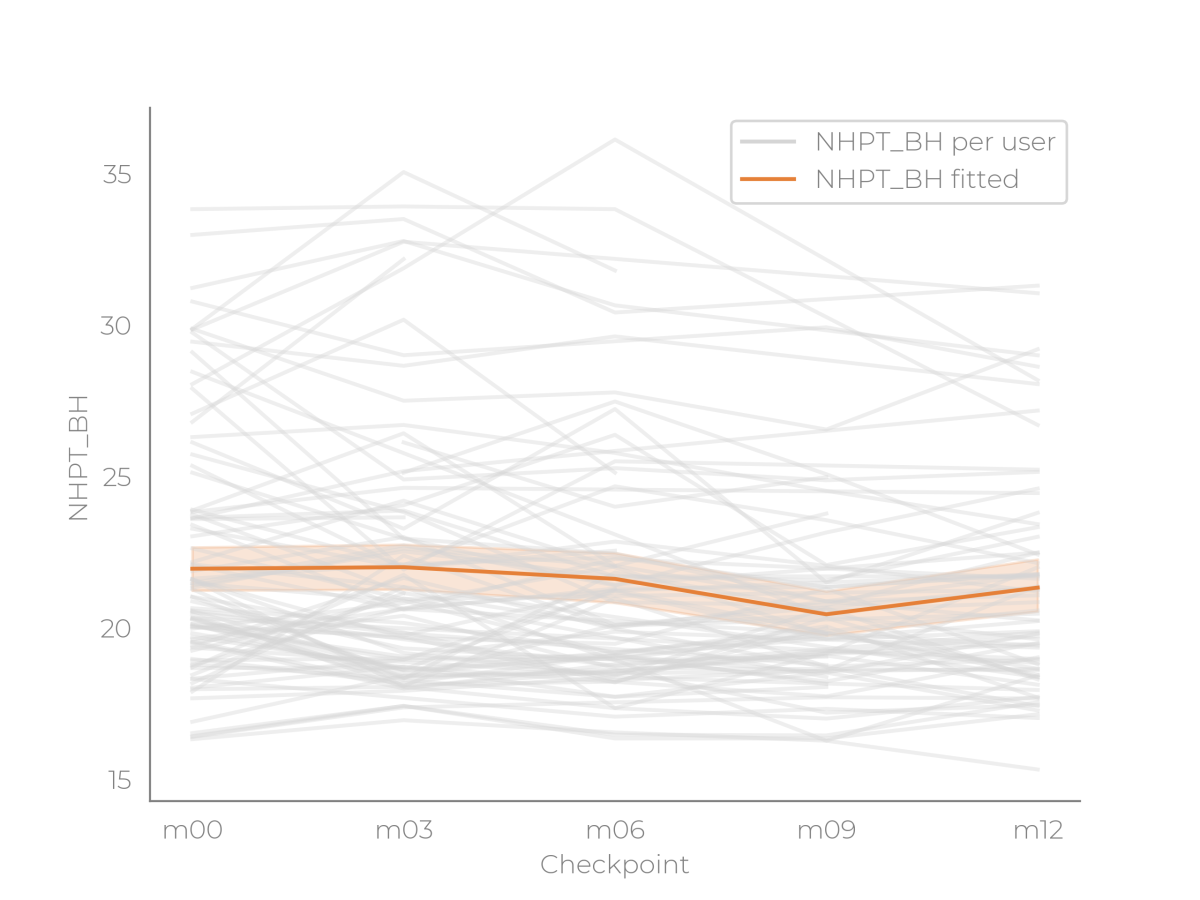

Supplement: Multimedia Appendix 4 [file jmir_v24i11e37614_app4.png]

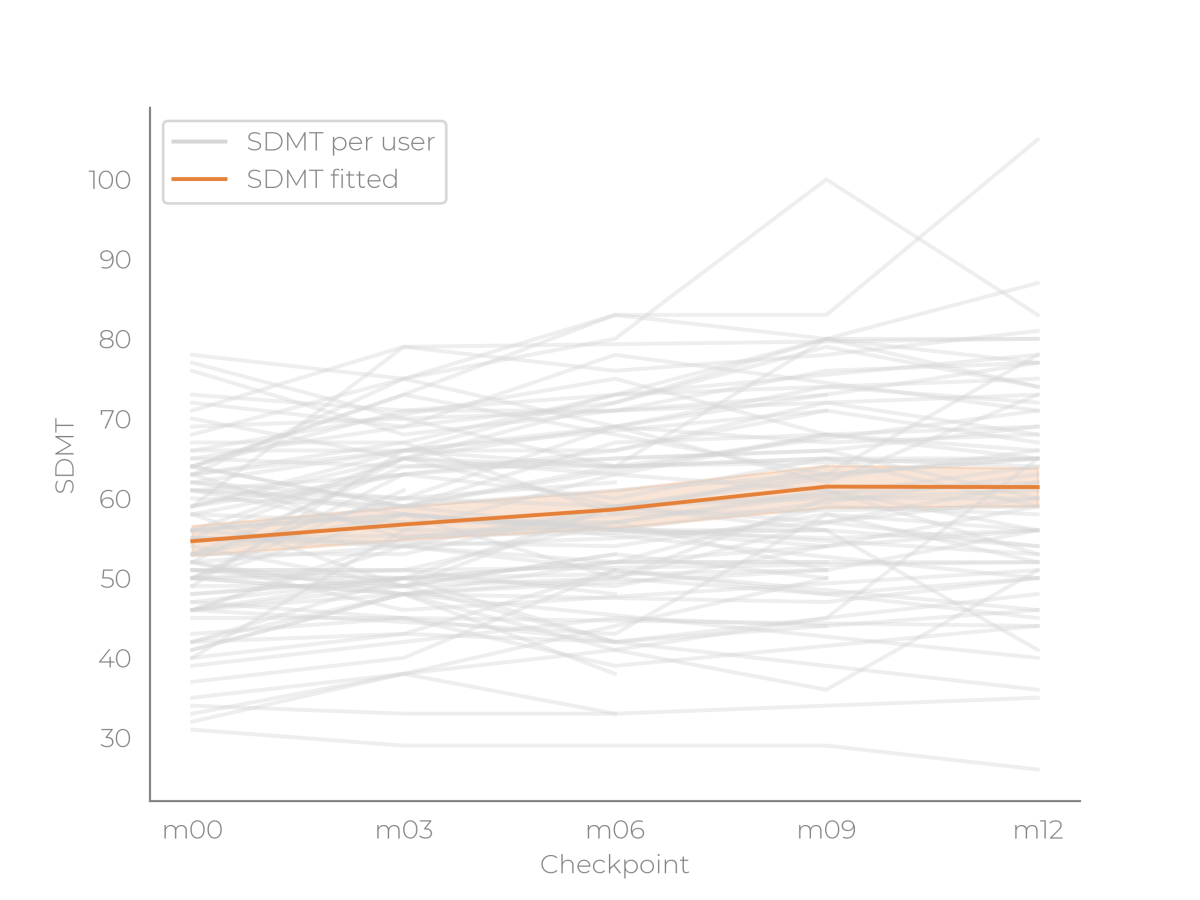

Supplement: Multimedia Appendix 5 [file jmir_v24i11e37614_app5.png]
